# Effects of various Agrobacterium rhizogenes strains on hairy root induction and analyses of primary and secondary metabolites in Ocimum basilicum

**DOI:** 10.3389/fpls.2022.983776

**Published:** 2022-10-17

**Authors:** Ramaraj Sathasivam, Minsol Choi, Ramalingam Radhakrishnan, Haejin Kwon, Jiwon Yoon, So Hwi Yang, Jae Kwang Kim, Yong Suk Chung, Sang Un Park

**Affiliations:** ^1^ Department of Crop Science, Chungnam National University, Daejeon, South Korea; ^2^ Department of Smart Agriculture Systems, Chungnam National University, Daejeon, South Korea; ^3^ Department of Botany, Jamal Mohamed College (Autonomous), Affiliated to Bharathidasan University, Tiruchirappalli, TN, India; ^4^ Division of Life Sciences and Convergence Research Center for Insect Vectors, College of Life Sciences and Bioengineering, Incheon National University, Incheon, South Korea; ^5^ Department of Plant Resources and Environment, College of Applied Life Sciences, Jeju National University, Jeju-si, South Korea

**Keywords:** agrobacterium rhizogenes, basil, hairy root, primary metabolites, secondary metabolites

## Abstract

The hairy root (HR) culture system is an excellent alternative strategy to the whole plant system for producing valuable compounds. However, selection of suitable *Agrobacterium* strain for the successful induction of HR is an essential step for enhanced production of beneficial secondary metabolites. In this study, we examined the transformation efficiency of various *A. rhizogenes* strains (ATCC 13333, ATCC 15834, A4, R1000, R1200, and R1601) for transgenic HRs induction in *Ocimum basilicum*. Among the tested strains, the R1601 was found to be one of the most promising strain for mass production of HR in terms of transformation efficiency (94%) and the number and length of HR (8.4 ± 0.52 and 1.68 ± 0.14 cm). The HR induced by the same strain exhibited highest levels of rosmarinic acid level (62.05 ± 4.94 µg/g DW) and total phenolic content (62.3 ± 4.95 µg/g DW). A total of 55 metabolites were identified using high-performance liquid chromatography (HPLC) and gas chromatography–time-of-flight mass spectrometry (GC-TOFMS). The PCA and PLS-DA plot of the identified metabolites showed that HR induced by A4 and ATCC 15834 displayed variation in primary and secondary metabolite contents. Analysis of the metabolic pathway identified a total of 56 pathways, among which 35 were found to be impacted. A heat map and hierarchical clustering analysis indicated that HR induced by different *Agrobacterium* strains exhibited differential metabolites profiles. In conclusion, *Agrobacterium* strains R1601 is one of the best and most promising strains for inducing mass HR production and enhanced levels of secondary metabolites in *O. basilicum*.

## Introduction

*Ocimum basilicum* L. is a perennial herb that belongs to the Lamiaceae family and is commonly called basil (Kwon et al., 2019; Mehring et al., 2020; Kwon et al., 2021). It is an annual aromatic herb, and an economically significant crop because of its wide range of biological and pharmacological properties (Irondi et al., 2016; Ezeani et al., 2017; Sestili et al., 2018; Bae et al., 2020; Zhan et al., 2020). It is widely grown in various countries such as Africa, Asia, India, Iran, South America, and other tropical countries in Asia (Kwon et al., 2019). In the past, basil was used to prevent cardiovascular-related diseases, and as an antispasmodic, digestive, stomachic, carminative, and tonic agent (Kwon et al., 2019). Basil extract contains phenolic and flavonoid compounds, such as cinnamic acid, caffeic acid, ferulic acid, rosmarinic acid, and sinapic acid (Marwat et al., 2011; Ghasemzadeh et al., 2016; Srivastava et al., 2016a; Pandey et al., 2022). Several previous studies have reported that basil extract has various biological properties, such as anti-aging, anticancer, antifungal, antihypertensive, antimicrobial, antioxidant, antiviral, nematocidal, and insect-repellent activities.

Phytochemical content has a significant impact on human health; however, in nature, obtaining the highest number of phytochemicals remains challenging. In plants, only specific tissues can synthesize and accumulate secondary metabolites (SMs) (Fang et al., 2012; Shi et al., 2020). Numerous biological techniques have been developed to increase the yield of plant compounds (Bourgaud et al., 2001; Nagella et al., 2013). Among these techniques, *Agrobacterium-*mediated transformation has been extensively used for hairy root (HR) culture production because it is commonly used for genetic manipulation, investigation of plant metabolic pathways, and the high production of various SMs (Georgiev et al., 2012; Bansal et al., 2014; Srivastava et al., 2016a; Kwon et al., 2021). *Agrobacterium rhizogenes* is a soil-borne, aerobic, rod-shaped bacterium, causative agent of HR disease in plants (Tzfira et al., 2004). *A. rhizogenes* carrying HR-inducing Ri plasmid can be used to transfer a specific DNA region, termed transfer DNA (T-DNA), into the plant genome to induce HR in plants (Park et al., 2017a; Yang et al., 2020; Sharma et al., 2021). The HRs that emerged after infection with *A. rhizogenes* showed high genetic and biosynthetic stability, and have been comprehensively used for the stable induction and increase of pharmaceutically important compounds (Gai et al., 2015; Thwe et al., 2016). *A. rhizogenes* is a suitable strain for producing valuable SMs owing to its improved growth regulators and is categorized by enhancing growth (Bansal et al., 2014; Tian, 2015; Thwe et al., 2016; Tavassoli and Safipour Afshar, 2018).

Recently, several molecular approaches have been used to enhance or regulate the genes involved in metabolic pathways that are necessary for the production of SMs (Georgiev et al., 2012; Sharma et al., 2013). In addition, HR is considered a potential source for the synthesis of new natural compounds. For instance, in the HR cultures of *Glycyrrhiza glabra and Datura stramonium* a novel antimicrobial compound (licoagrodione) and a tropane alkaloid ester was identified (Li et al., 1998; Berkov et al., 2003). Importantly, for rapid HR growth, phytohormones are not required for cultivation (Kwon et al., 2021). Hence, HR culture is a valuable tool for the isolation and development of several novel pharmacologically active compounds (Thwe et al., 2016).

Recently, different strains of *A. rhizogenes* have been extensively used for plant transformation. Previous studies have reported that *A. rhizogenes* can influence the genes that participate in plant SM production (Ooi et al., 2013; Sudha et al., 2013). Hence, the identification and selection of a suitable strain of *Agrobacterium* to obtain transformed HR lines is considerably dependent on the plant species that need to be investigated experimentally. The morphology, virulence, and growth rate variations might be related to the root-inducing Ri plasmid within the bacterial strain (Thwe et al., 2016; Gutierrez-Valdes et al., 2020). In the Ri plasmid, the T-DNA genes and *rol* oncogenes showed salient changes in phenotypic and biochemical characteristics of the transformed HR. The *rol* gene is responsible for activating SMs in the transformed cells of the Araliaceae, Rosaceae, Rubiaceae, Solanaceae, and Vitaceae families (Bulgakov et al., 2008; Thwe et al., 2016).

Previous studies have reported that the HR induced by different strains of *Agrobacterium* displayed differential accumulation of rosmarinic acid, betulin, and betulinic acid in *Agastache rugosa* and *Morus alba*, respectively (Park et al., 2017a; Park et al., 2017b). Thwe et al., 2016 reported that different *A. rhizogenes* strains induce different levels of HR and SM production, which lead to differential expression of phenylpropanoid pathway genes in *Fagopyrum tataricum*. In addition, there are several studies on the *O. basilicum* HR induction by using different *A. rhizogenes* strains and analysis of rosmarinic acid content (Tada et al., 1996; Bais et al., 2002; Srivastava et al., 2016a; Srivastava et al., 2016b; Srivastava et al., 2019; Abadi et al., 2020; Kwon et al., 2021; Pandey et al., 2022). However, in these studies, the primary and secondary metabolites were not analyzed simultaneously. Additionally, to date, no studies have been conducted on the transformation of *O. basilicum* infection with different strains of *A. rhizogenes* for phenylpropanoid accumulation and metabolic profiling of primary metabolites. As a novel study, this work was aimed to induce HRs in *O. basilicum* by different strains of *A. rhizogenes* for metabolomics profiling of primary and secondary metabolites of pharmacological importance. The results of this study will provide immense knowledge on the selection and identification of a suitable strain of *A. rhizogenes* to improve the yield of high-commercial-value compounds such as amino acids, organic acids, carbohydrates, and phenolic compounds in basil. In addition, this study could be scaled up by using identified *A. rhizogenes* strain for enhanced production of secondary metabolites.

## Materials and methods

### Sterilization of seeds and germination

Seeds of *O. basilicum* were obtained from Danong Seed Co., Namyangju, Korea. The seeds were surface-sterilized with 70% ethanol and 4% sodium hypochlorite (NaClO) for 2 and 4 min, respectively. The seeds were then washed with sterile deionized water three times and dried with sterile tissue paper. Murashige and Skoog (MS) basal agar (0.7% w/v) medium (Murashige and Skoog, 1962) was prepared and the pH was adjusted to 5.8 before autoclaving. After cooling, the medium was poured into a petri plate. Approximately, ten seeds were placed on a petri plate and then placed into a growth chamber at 25°C under standard white fluorescent light with 16-h dark/8-h light conditions with a photon flux density ca. 35 µmol s^-1^ m^-2^.

### Bacterial strains and culture conditions

To determine the transformation efficiency, six different strains of *A. rhizogenes* (ATCC 13333, ATCC 15834, A4, R1000, R1200, and R1601) were used for the experiment. The strains were obtained from the Centro de Investigacion Cientifica de Yucatan, Mexico, and conserved in our laboratory. All culture strains were maintained at –80°C in glycerol stock in our laboratory. The viability of each strain was determined by occasional growth on Luria Broth (LB) agar. Each *A. rhizogenes* strain was cultured in a 100-ml flask containing 30 ml of LB broth and incubated at 28°C in a shaking chamber (180 rpm) overnight or until the culture reached the mid-log phase (OD_600_ = 0.5). The cells were harvested by centrifugation at 4000 rpm and washed with phosphate-buffered saline (pH 7.2), and resuspended in liquid MS medium. For the inoculation of *A. rhizogenes* strains, the cell density was adjusted to OD_600_ = 1.0.

### Hairy root culture induction

The grown leaves of *O. basilicum* were excised from sterile *in vitro* plantlets, and the leaves were cut at the ends to produce 7 × 7 mm sections. The leaves were immersed in the media containing *A. rhizogenes* for 10 min and then dried with sterile tissue paper. The explants were placed on MS agar medium and incubated in the dark for two days at 25°C. The explants were harvested, washed with sterile deionized water, and transferred to a solid half-strength MS medium containing cefotaxime (500 mg/L). After 7 days, the HRs were transferred to an antibiotic-free medium under aseptic conditions, and promptly growing HRs were aseptically attained. Approximately 0.5 g well-grown HRs were transferred to a liquid MS medium (30 ml), and then the cultures were incubated for 28 days on an orbital shaker (100 rpm) at 25°C. The HRs were collected, immediately flash-frozen using liquid nitrogen, and stored at –80°C until further analysis.

### Genomic DNA extraction and PCR analysis

Genomic DNA of the transformed basil HRs was extracted using a DNeasy Plant Mini Kit (Qiagen, Valencia, CA, USA). Transgenic HRs were confirmed by amplifying the four *rol* genes (A and B). Primers for the *rol* genes were obtained from a previous study (Thwe et al., 2016). PCR conditions and analysis of fragment sizes were performed according to the protocol described by Thwe et al. (2016).

### HPLC analysis

To estimate the phenolic compounds present in the transformed *O. basilicum*, 0.1g of each finely powdered HR sample was collected, 3 ml of 100% methanol was added, and then kept in a sonicator for 1 h at 60°C. The mixture was then gently mixed for 20 min. After incubation, the mixture was centrifuged at 10,000 rpm for 10 min. The supernatant was removed, and the filter was sterilized through a 0.45 μm PTFE syringe filter (Toyo Roshi Kaisha, Ltd., Japan). Phenolic compounds were separated using an Agilent Technologies HPLC 1200 series (Palo Alto, CA, USA) coupled with a C_18_ column (250×4.6 mm, 5 μm) at 280 nm. The column temperature, mobile phase, HPLC conditions, gradient programs, and quantification of phenolic compounds were similar to those described previously by Yeo et al. (2021). All the standards were purchased from Sigma-Aldrich Co., Ltd. (St. Louis, MO, USA) and the purity of each standard was as follows; 4-hydroxybenzoic acid (≥99%), chlorogenic acid (≥95%), caffeic acid (≥98%), *p*-coumaric acid (≥98%), and rosmarinic acid (≥97%).

### GC-TOFMS

To quantify the hydrophilic compounds present in the transformed *O. basilicum*, 10 mg of each finely powdered HR sample was collected and 1 ml of CHCl_3_/MeOH/water (1:2.5:1 *v*/*v*/*v*) was added. As an internal standard 60 µL of 0.2 g L^−1^ adonitol was added. Extraction of the hydrophilic compounds was carried out at 37°C in a thermomixer at a mixing speed of 1200 rpm for 3 min, followed by centrifugation for 5 min at 16,000× g to separate the polar phase present in the supernatant. Transfer 0.8 ml of polar phase into a new 2 ml centrifuge tube and, 0.4 ml of sterile deionized water was added. After gentle mixing, the mixture was centrifuged at 16,000× g for 5 min to separate the polar phase and then evaporated for 3 h in a CVE-2000 centrifugal concentrator (Daejeon, Korea). The remaining deposit was lyophilized using a freeze dryer for 15 h. Furthermore, lyophilized materials were subjected to two steps: Step 1 was the addition of 80 μL of 20 g·L^−1^ methoxyamine hydrochloride/pyridine, and shaking at 30°C for 90 min. Step 2 involved the addition of *N*-methyl-*N*-(trimethylsilyl) trifluoroacetamide and heating for 30 min at 37°C. After filter sterilization, the product was injected into an Agilent 7890 GC system (Agilent, Atlanta, GA, USA) for analysis. Operating conditions, flow rate, gradient program, and metabolite identification were performed according to the protocol described by Park et al. (2020).

### Statistical analysis

Statistical analysis of the data was performed using the statistical analysis system 9.4 (SAS Institute, Inc., Cary, NC, USA). All values are presented as the mean ± standard deviation of three biological replicates. Data were analyzed by analysis of variance (ANOVA) using Duncan’s multiple range test (*p* < 0.05). Heatmap, principal component analysis (PCA), partial least-squares discriminant analysis (PLS-DA), Pearson correlation analysis, hierarchical clustering, pathway impact, and variable importance in projection (VIP) for the identified metabolites were performed using the free online software MetaboAnalyst 5.0, with auto-scaling.

## Results

### Hairy root culture induction

**Figure 1:**
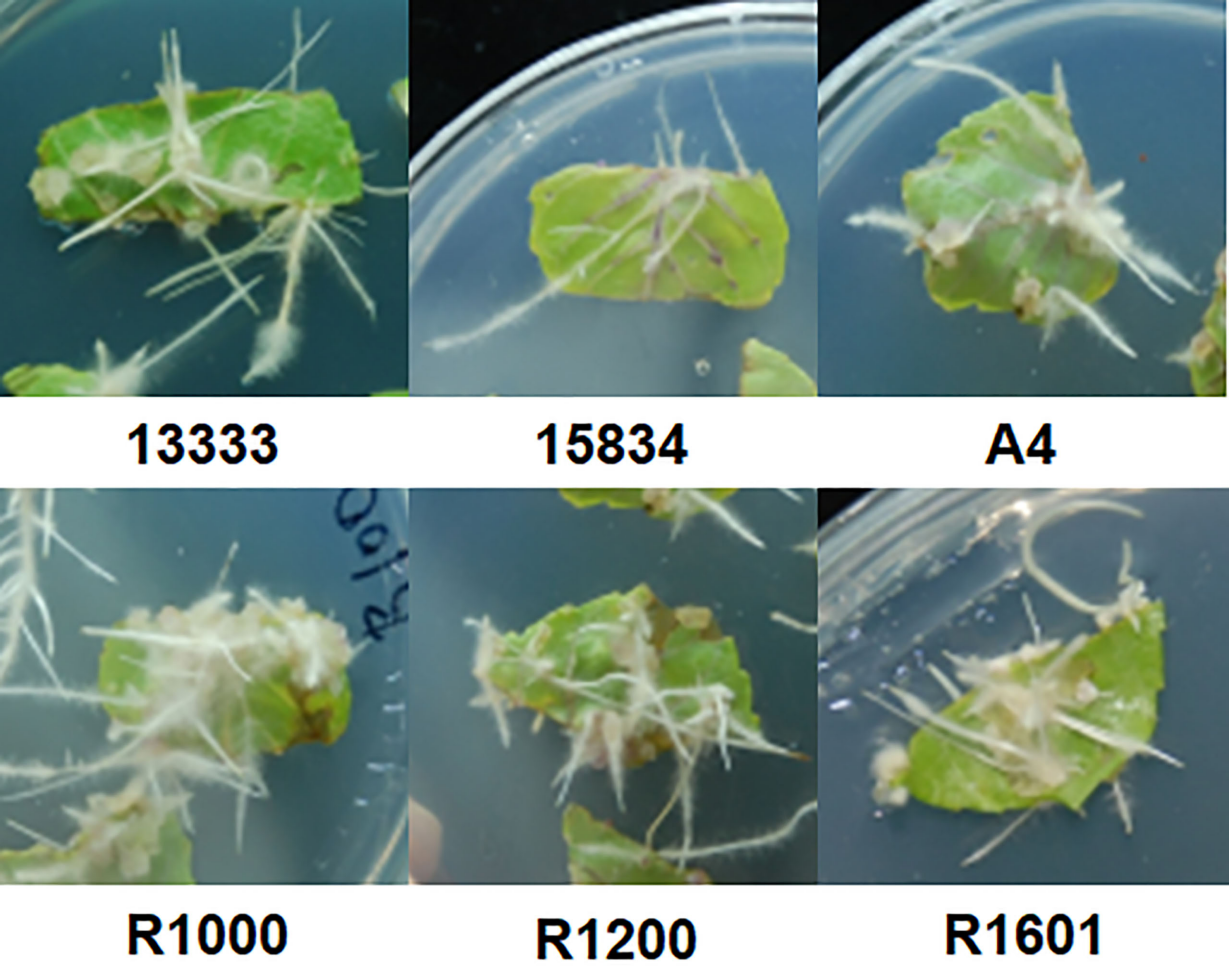
Hairy root induction from the leaves of *O. basilicum*.

**Figure 2:**
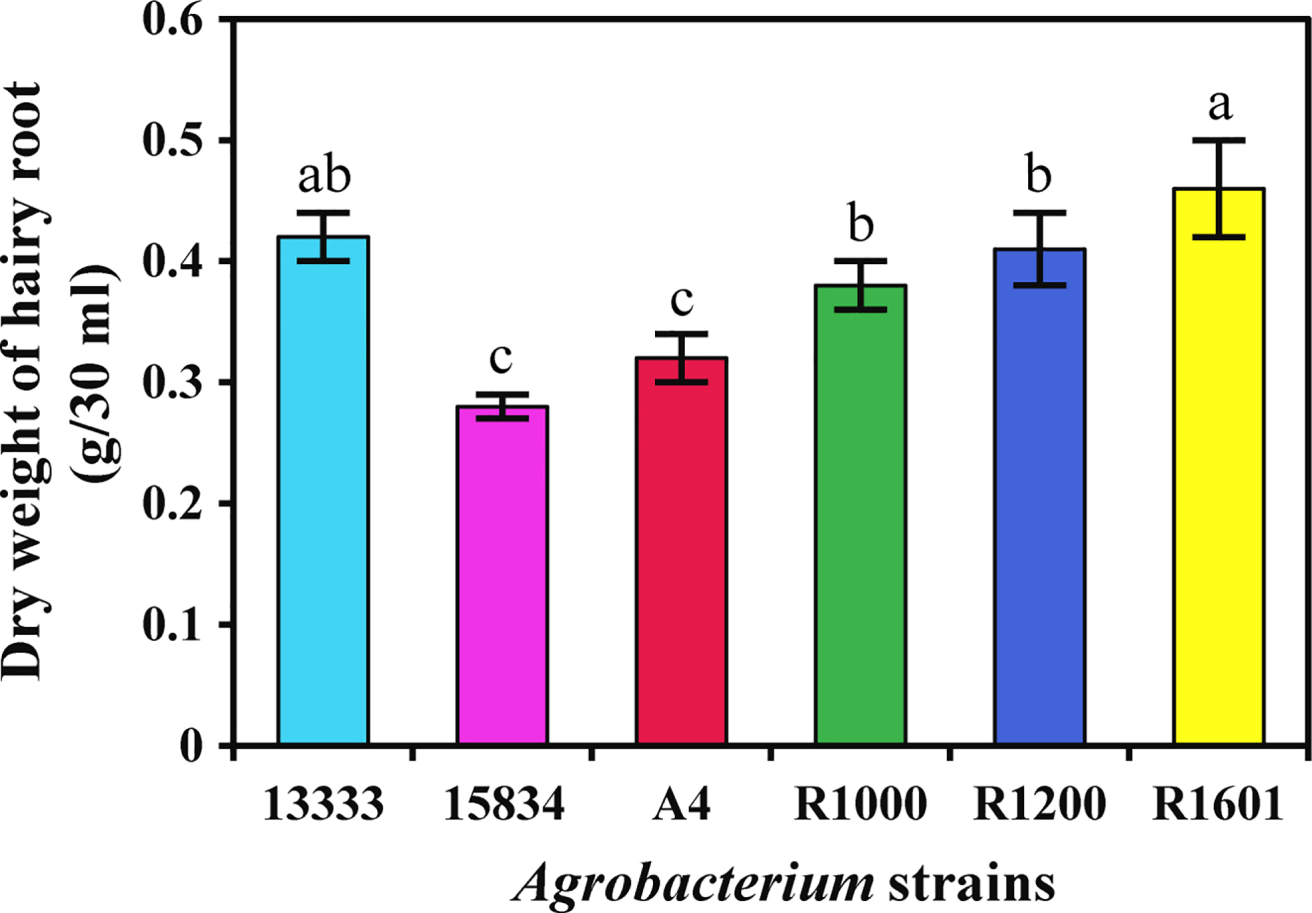
Dry weight of *O. basilicum* hairy root (g/30 ml medium) induced by different *Agrobacterium rhizogenes* strains. The data were obtained after 14 days of culturing in MS liquid medium. Data were obtained from three biological replicates. Different letters indicate the significant difference at p < 0.05 by Duncan’s multiple range tests.

The isolated *A. rhizogenes* strains ATCC 13333, ATCC 15834, A4, R1000, R1200, and R1601, which induces HR in plants, were used to analyze transformation efficiency in *O. basilicum*. Wounded leaves of *O. basilicum* were infected with different *A. rhizogenes* strains to develop an HR culture system (**Figure 1**). The results showed that different *A. rhizogenes* strains exhibited different infection efficiency percentages, numbers of HRs, and lengths of HRs in *O. basilicum*. Among the different strains, the highest infection efficiency percentage was obtained for strain R1601 (94%), whereas the lowest was attained for the ATCC 15834 strain (66%). The R1000, R1200, ATCC 13333, and A4 strains showed infection efficiencies of 93, 91, 91, 73, and 66, respectively (**Table 1**). Each strain showed a slight difference in the number of HRs per explant and the HR length. Strain R1601 showed the highest number of HRs per explant and HR length (average, 8.4 and 1.68 cm), followed by ATCC 13333, R1000, A4, R1200, and ATCC 15834. The necrotic explant tissues in the form of HRs on MS medium were fast and exhibited different transformation efficiencies for each strain. In addition, the induction of HR was fast. The dry weight of the HRs obtained showed that R1601 (0.46 g) was the highest, followed by ATCC 13333 (0.42 g), R1200 (0.41 g), R1000 (0.38 g), and A4 (0.32 g), whereas ATCC 15834 (0.28 g) showed the lowest dry weight (**Figure 2**). From these results, it was inferred that R1601 showed the highest percentage of infection efficiency, number and length of HRs per explant, and dry weight of roots.

**Table 1:** Effects of different *Agrobacterium rhizogenes* strains on hairy root growth of *O. basilicum*.

Agrobacterium strain	Transformation efficiency (%)	Number of hairy roots/explants	Length of hairy root (cm)
ATCC 13333	91	6.3 ± 0.48b	1.65 ± 0.14a
ATCC 15834	66	3.6 ± 0.28d	1.48 ± 0.16a
A4	73	5.2 ± 0.21c	1.57 ± 0.23a
R1000	93	7.7 ± 0.42a	1.63 ± 0.16a
R1200	91	8.1 ± 0.65a	1.55 ± 0.12a
R1601	94	8.4 ± 0.52a	1.68 ± 0.14a

Different alphabetical letters a-d denote significant differences (p < 0.05).

### Analysis of phenolic compounds

In total, five individual phenolic compounds were quantified using HPLC in the HRs of *O. basilicum* (**Table 2**). HPLC results showed a slight variation in the phenolic compound content of all HR obtained with different *A. rhizogenes* strains. The highest individual phenolic content was achieved with rosmarinic acid, whereas the lowest was obtained with 4-hydroxybenzoic acid and *p*-coumaric acid. The rosmarinic acid content was significantly higher in all HR samples induced by different strains in the following orders: R1601 (62.05 ± 4.94 µg/mg DW), R1200 (60.66 ± 2.60 µg/mg DW), ATCC 13333 (59.34 ± 1.95 µg/mg DW), R1000 (50.50 ± 3.44 µg/mg DW), A4 (49.08 ± 1.09 µg/mg DW), and ATCC 15834 (48.58 ± 2.12 µg/mg DW). Interestingly, 4-hydroxybenzoic acid was not detected in the HR sample induced by R1200 only, whereas *p*-coumaric acid was not detected in the HR sample induce by R1601 and ATCC 15834. Caffeic acid content showed the second-highest accumulation with 0.25, 0.19, 0.19, 0.19, 0.18, and 0.16 (µg/mg DW), in the HR sample induced by ATCC 15834, R1601, R1200, R1000, A4, and ATCC 13333, respectively. The chlorogenic acid content was slightly higher than that of 4-hydroxybenzoic acid and *p*-coumaric acid. Total phenolic content was found in the R1601 infected strain (62.29 ± 4.95 µg/mg DW), which was 1.27-, 1.05-, 1.22-, 1.26-, and 1.02-fold higher than that in the ATCC 15834, ATCC 13333, R1000, A4, and R1200 strains, respectively. The results showed that, among the different *A. rhizogenes* infections, R1601 was the most important strain for the induction of phenolic compounds in *O. basilicum*.

**Table 2:** Phenolic acid content in the induced hairy roots of *O. basilicum* by various *Agrobacterium rhizogenes* strains (µg/mg DW).

Phenolic compounds	ATCC 13333	ATCC 15834	A4	R1000	R1200	R1601
4-hydroxybenzoic acid	0.01 ± 0.00a	0.01 ± 0.00a	0.01 ± 0.00a	0.01 ± 0.00a	ND	0.01 ± 0.00a
Chlorogenic acid	0.06 ± 0.00a	0.03 ± 0.01c	0.05 ± 0.01ab	0.05 ± 0.00ab	0.05 ± 0.00ab	0.04 ± 0.00bc
Caffeic acid	0.16 ± 0.00d	0.25 ± 0.00a	0.18 ± 0.00c	0.19 ± 0.00b	0.19 ± 0.00b	0.19 ± 0.01b
*p*-coumaric acid	0.01 ± 0.00a	ND	0.01 ± 0.00a	0.01 ± 0.00a	0.01 ± 0.00a	ND
Rosmarinic acid	59.34 ± 1.95a	48.58 ± 2.12b	49.08 ± 1.09b	50.50 ± 3.44b	60.66 ± 2.60a	62.05 ± 4.94a
Total	59.58 ± 1.95a	48.87 ± 2.13b	49.33 ± 1.1b	50.76 ± 3.44b	60.91 ± 2.60a	62.29 ± 4.95a

Different alphabetical letters a-d denote significant differences (p < 0.05).

ND, Not Detected.

### Metabolic profiling of identified metabolites by using HPLC and GC-TOFMS

**Figure 3:**
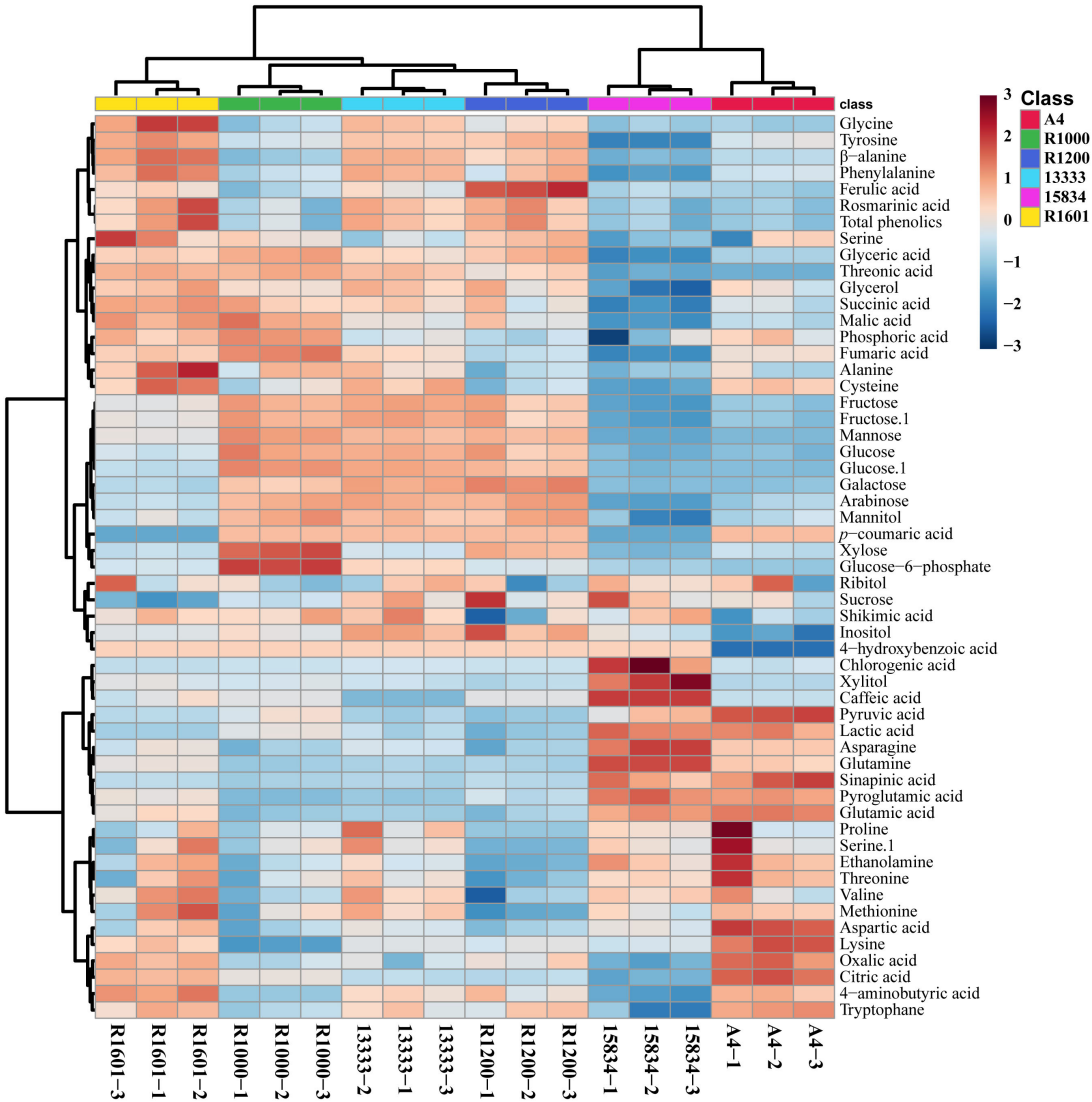
Heatmap representing the differences in relative metabolite concentrations in *O. basilicum* hairy root induced by different *Agrobacterium rhizogenes* strains. Increases and decreases in the metabolites are shown in red and blue color, respectively.

In total, 55 metabolites were identified and quantified from HRs induced by different *A. rhizogenes* infected strain (**Figure 3** and **Supplementary Figures S1-S6**). A heat map analysis of these metabolites showed that the level of tricarboxylic acid (TCA) cycle intermediates, such as citric acid, was highest in the A4 and R1601 strains, whereas fumaric acid was highest in the R1000, R1601, ATCC 13333, and A4 strains. The highest level of malic acid was observed in the HR samples induced by R1601 and R1000. The levels of organic acids, such as citric acid and oxalic acid, were highest in the A4 and R1601 infected strains. The highest contents of lactic acid, pyruvic acid, and sinapinic acid were achieved only in the HR samples induced by A4 and ATCC 15834 infected strains, compared to those in the other infected strains. Interestingly, the highest sugar level was observed in the HR samples induced by ATCC 13333, R1000, and R1200. However, the lowest level was achieved in HR samples induced by ATCC 15834, followed by A4, and R1601. Glyceric and threonic acid levels were highest in the HR samples induced by R1000, R1601, ATCC 13333, and R1200, whereas shikimic acid levels were highest in the HR samples induced by ATCC 13333, R1601, and R1000. Moreover, most amino acid levels were the highest in the HR samples induced by ATCC 13333, ATCC 15834, and A4. The levels of asparagine, glutamine, glutamic acid, and proglutamic acid were highest only in the HR samples induced by A4 and ATCC 15834, and phenylalanine and tyrosine levels were highest in R1601, ATCC 13333, and R1200. Significantly higher (p ≤ 0.05) individual metabolites in *O. basilicum* HRs induced by different *Agrobacterium rhizogenes* strains are shown in **Supplementary Figure S7**. These results show that different strains increased the content of specific metabolites. The results of this study may be helpful for researchers to select a particular *Agrobacterium* strain to increase the specific metabolite content in the HRs of *O. basilicum*.

**Figure 4:**
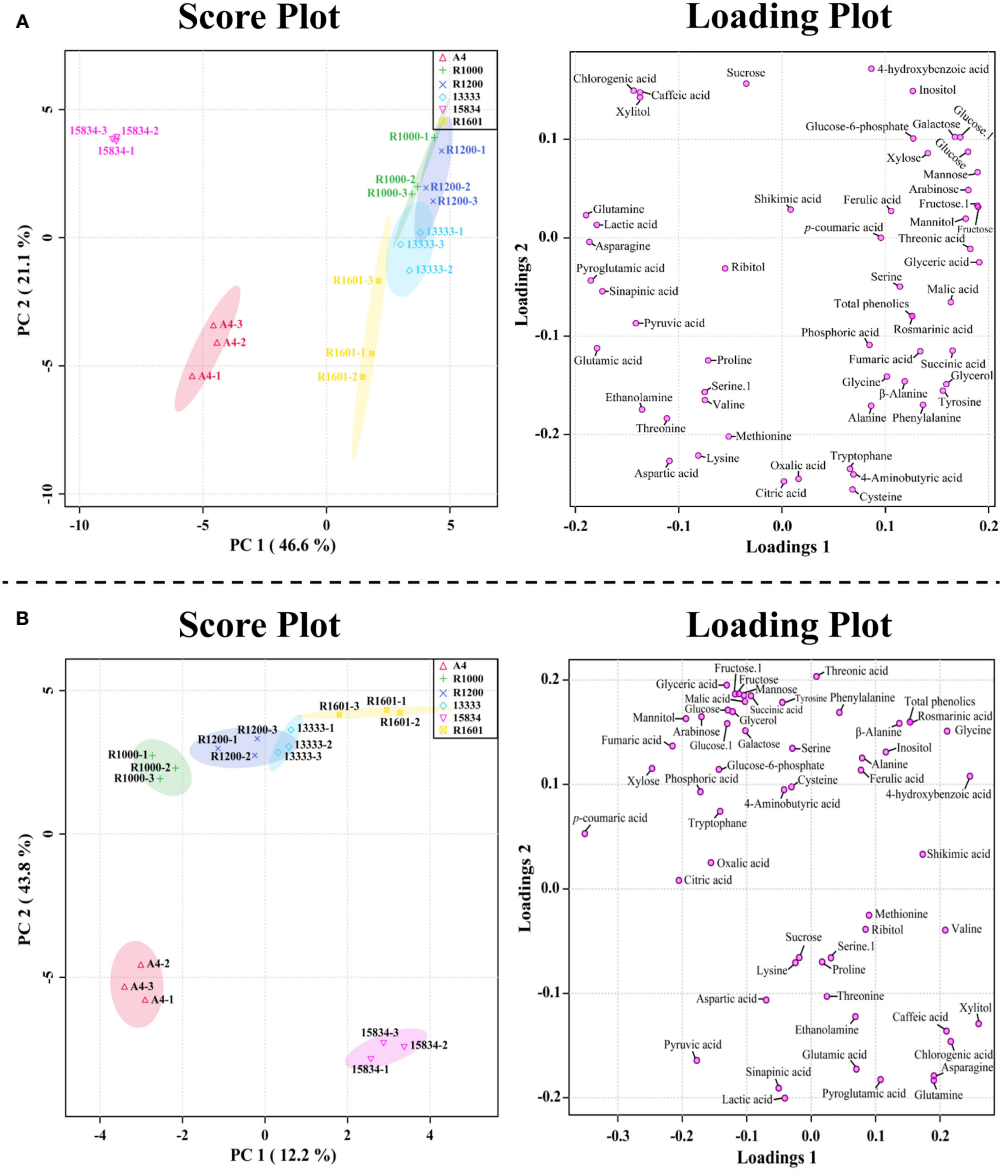
Score and loading plot of the **(A)** PCA model and **(B)** PLS-DA results obtained from the metabolites identified in *O. basilicum* hairy root induced by different *Agrobacterium rhizogenes* strains.

PCA results supported the above metabolic difference results obtained in different *A. rhizogenes* infected strains. The results of PCA showed a visualization of the metabolite data obtained from the different *A. rhizogenes* infected strains with two-component analysis (46.6% and 21.1% of the variance, respectively), as showed in **Figure 4**. Principal component 1 (PC1) showed that the A4 and ATCC 15834 infected HRs showed a clear separation, whereas other infected strains such as R1601, ATCC 13333, R1200, and R1000 did not separate well. A similar result was obtained through the PLS-DA analysis, where A4 and ATCC 15834 were separated when compared to other infected strains in its two-component analysis, describing 12.2% and 43.8% of the variance, respectively (**Figure 4**). This result supports the heatmap result which showed that most of the metabolites were highest in R1601, ATCC 13333, R1200, and R1000 which form a close group in the PCA and PLS-DA models (**Figure 4**).

**Figure 5:**
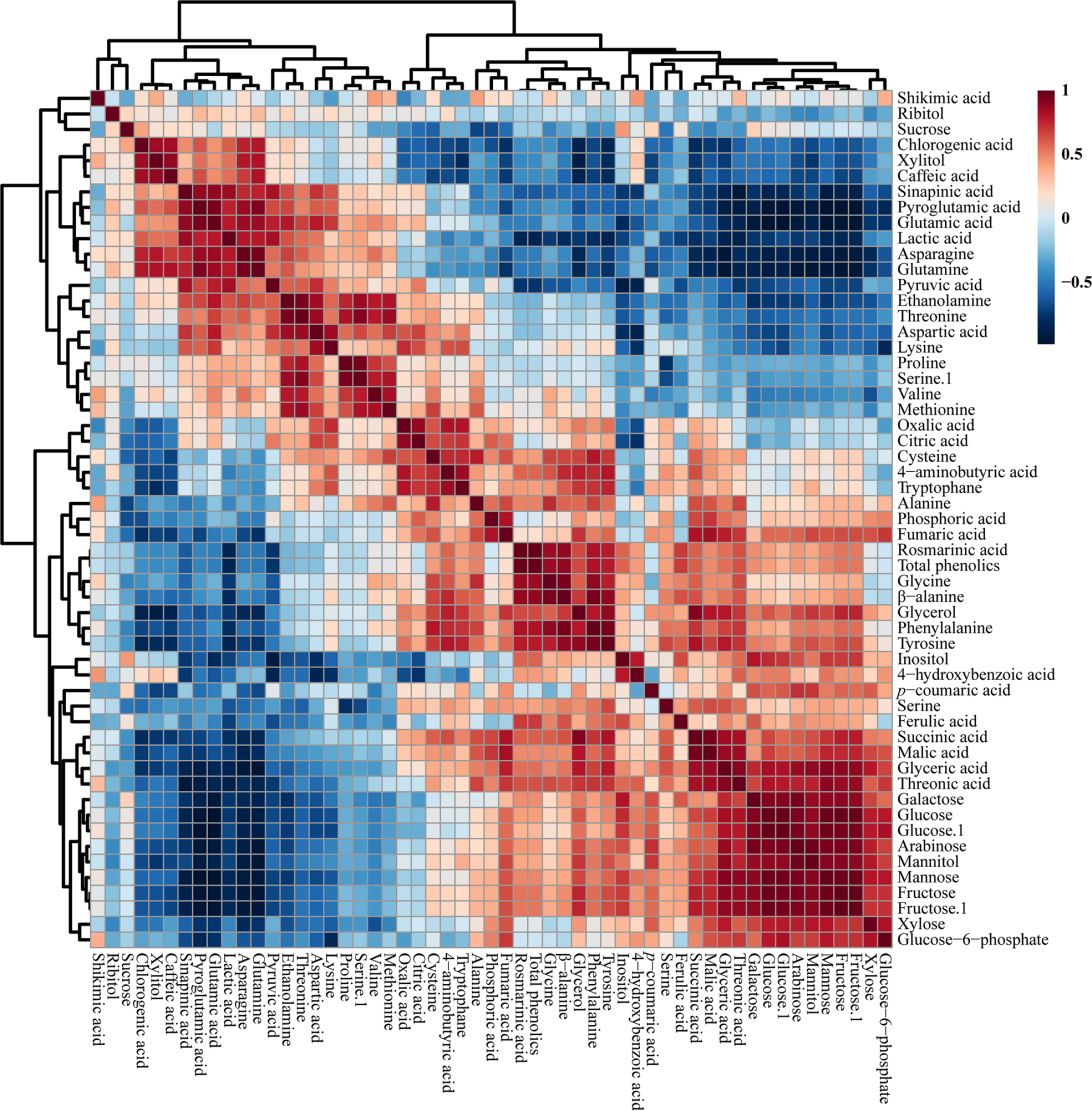
Correlation matrix of metabolites identified in *O. basilicum* hairy root induced by different *Agrobacterium rhizogenes* strains. Each colored box indicates the Pearson’s correlation coefficient for a pair of compounds, and the value of the correlation coefficient is represented by the intensity of the blue or red color, as indicated on the color scale.

The correlations between the identified metabolites in the HRs of *O. basilicum* infected with different *Agrobacterium* strains were examined using Pearson’s correlation (**Figure 5**). Phenylalanine synthesized by the shikimate biosynthetic pathway was positively correlated with shikimic acid. Phenylalanine is an initial step in the synthesis of phenolic compounds, which is strongly correlated with the total phenolic content (*r* = 0.813, *p* = 0.000). Similarly, phenylalanine showed a strong correlation with rosmarinic acid (*r* =0.814, *p* = 0.000), an intermediate compound in the phenylpropanoid biosynthetic pathway. Moreover, glucose 6-phosphate was positively correlated with fructose levels (*r* = 0.722, *p* = 0.000). Furthermore, TCA cycle intermediates such as fumaric acid (*r* = 542, *p* = 0.200) and malic acid (r = 0.350, p = 0.155) were positively correlated with citric acid. Sucrose was positively correlated with most phenolic compounds such as chlorogenic acid, caffeic acid, sinapic acid, 4-hydroxybenzoic acid, *p*-coumaric acid, and ferulic acid, and sugar compounds, such as xylitol, inositol, galactose, glucose, arabinose, and fructose.

**Figure 6:**
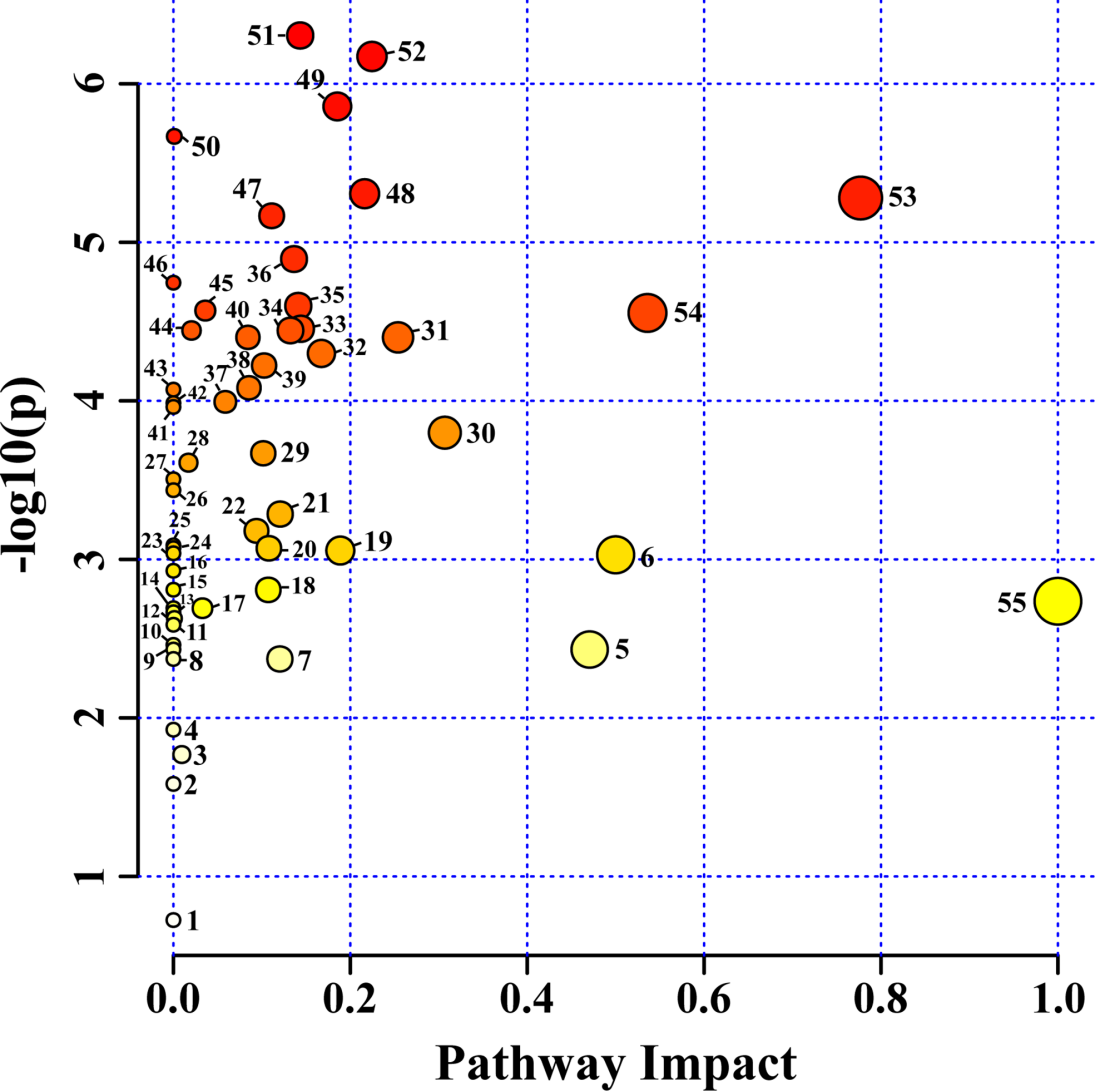
Identified metabolites and their pathway impact on *O. basilicum* hairy root induced by different *Agrobacterium rhizogenes* strains. 1) Sphingolipid metabolism; 2) Valine, leucine, and isoleucine degradation; 3) Glycerophospholipid metabolism; 4) Selenocompound metabolism; 5) Phenylalanine metabolism; 6) Isoquinoline alkaloid biosynthesis; 7) Tryptophan metabolism; 8) Indole alkaloid biosynthesis; 9) Tropane, piperidine and pyridine alkaloid biosynthesis; 10) Nicotinate and nicotinamide metabolism; 11) Porphyrin and chlorophyll metabolism; 12) Fructose and mannose metabolism; 13) Propanoate metabolism; 14) Ascorbate and aldarate metabolism; 15) Terpenoid backbone biosynthesis; 16) Nitrogen metabolism; 17) Phosphatidylinositol signaling system; 18) Valine, leucine and isoleucine biosynthesis; 19) Cysteine and methionine metabolism; 20) Amino sugar and nucleotide sugar metabolism; 21) Glycolysis/Gluconeogenesis; 22) Sulfur metabolism; 23) Purine metabolism; 24) Lysine degradation; 25) Lysine biosynthesis; 26) Monobactam biosynthesis; 27) Glucosinolate biosynthesis; 28) Glycerolipid metabolism; 29) Phenylalanine, tyrosine and tryptophan biosynthesis; 30) Starch and sucrose metabolism; 31) Beta-alanine metabolism; 32) Pyruvate metabolism; 33) Arginine and proline metabolism; 34) Stilbenoid, diarylheptanoid and gingerol biosynthesis; 35) Glutathione metabolism; 36) Butanoate metabolism; 37) Galactose metabolism; 38) Arginine biosynthesis; 39) Inositol phosphate metabolism; 40) Pantothenate and CoA biosynthesis; 41) Pyrimidine metabolism; 42) Thiamine metabolism; 43) Pentose and glucoronate interconversions; 44) Flavonoid biosynthesis; 45) Carbon fixation in photosynthetic organisms; 46) Cyanomino acid metabolism; 47) Aminoacyl-tRNA biosynthesis; 48) Tyrosine metabolism; 49) Citrate cycle (TCA cycle); 50) Ubiquinone and other terpenoid-quinone biosynthesis; 51) Phenylpropanoid biosynthesis; 52) Glyoxylate and dicarboxylate metabolism; 53) Alanine, aspartate, and glutamate metabolism; 54) Glycine, serine, and threonine metabolism; 55) Biosynthesis of secondary metabolites-unclassified.

Fifty-six pathways were identified in the HRs of *O. basilicum* infected with different *Agrobacterium* strains. For pathway analysis, *Arabidopsis thaliana* Kyoto Encyclopedia of Genes and Genomes (KEGG) metabolic pathway was used as a source for pathway libraries (**Figure 6**). Among these, 35 pathways were found to be impacted in this study (**Supplementary Table S1**). Furthermore, six of pathways: 1) alanine, aspartate, and glutamate metabolism, 2) arginine biosynthesis, 3) arginine and proline metabolism, 4) tyrosine metabolism, 5) phenylpropanoid biosynthesis, 6) glycine, serine, and threonine metabolism, are related to amino acid biosynthesis and metabolism. Similarly, six carbohydrate metabolism pathways were also affected: 1) inositol phosphate metabolism, 2) pyruvate metabolism, 3) galactose metabolism, 4) glyoxylate and dicarboxylate metabolism, 5) TCA cycle, 6) starch metabolism, and sucrose metabolism. Isoquinoline alkaloid biosynthesis, butanoate metabolism, glutathione metabolism, and primary biosynthetic pathways were also affected. These results suggest that various nutraceutical pathways have been affected.

**Figure 7:**
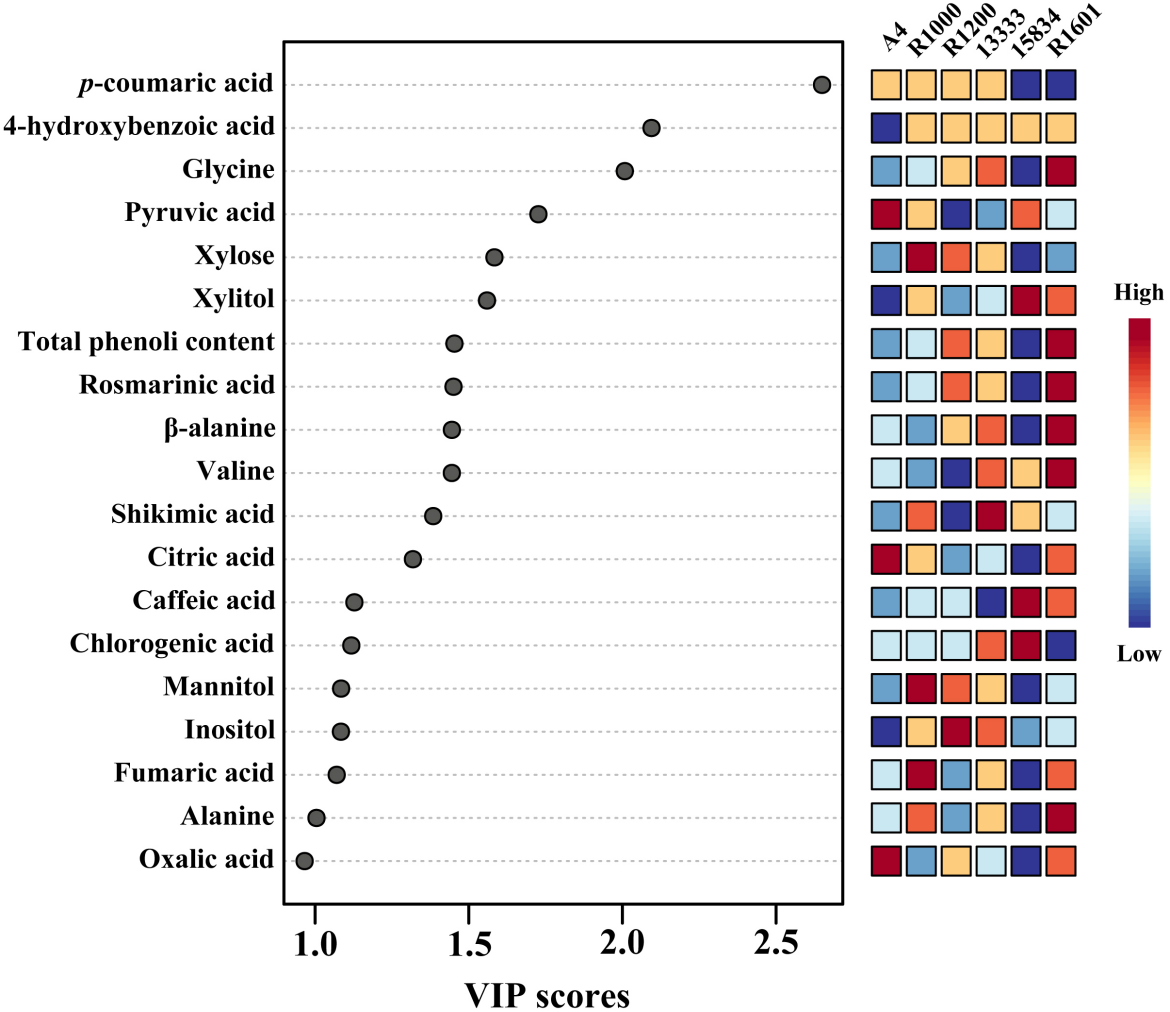
The key components, separating the *O. basilicum* hairy root induced by different *Agrobacterium rhizogenes* strains, are based on the VIP scores obtained through the PLS-DA model.

To identify the important metabolites among the various *Agrobacterium* infected strains, we performed variable importance in projection (VIP) and SAM analyses. PLS-DA recognized the most important metabolites based on a VIP value of the five-component model greater than 1. In total, 19 compounds were identified as important metabolites (VIP > 1) among the various *Agrobacterium* infected strains (**Figure 7**). In addition, SAM identified similar metabolites based on the d-value of the metabolites at a delta value of 0.1, a false discovery rate of 0.023, and a false positive of 32.85 (**Supplementary Figure S8**).

## Discussion

Functional compounds and metabolites from plants have received considerable attention owing to their desired pharmaceutical activities and various applications in the medicinal field. *O. basilicum* is an important traditional medicinal plant because of the presence of phenolic and flavonoid compounds. Hence, selecting and optimizing the exact and most reliable transformation methods and strains are important for increasing the content of phenolic, flavonoid, and phytochemical compounds in *O. basilicum*. *A. rhizogenes* and *A. tumefaciens* play vital roles in plant transformation by increasing the production of various novel compounds. As mentioned in the introduction, the induction of HRs using *A. rhizogenes* has several advantages over cell and callus cultures owing to the high stability of the gene and reduced cultivation costs.

Several studies have analyzed the effect of different strains of *A. rhizogenes* on the growth and development of HR lines and SM production. Ionkova et al. (1997) studied that infection of *Astragalus mongholics* with different *A. rhizogenes* strains exhibiting different growth and saponin contents. In transgenic HRs of *Hyoscyamus muticus*, different *Agrobacterium* strains significantly influenced growth rate and tropane accumulation (Vanhala et al., 1995; Mateus et al., 2000). In addition, the HR culture system for *Rubia akane* developed by transformation with five different *Agrobacterium* strains showed different HR infection efficiencies and anthraquinone accumulation (Lee et al., 2010). Similarly, in *M. alba*, the induction of HR by the transformation of different *A. rhizogenes* strains resulted in varied infection efficiency, number and length of HRs, dry weight of roots, and betulin and betulinic acid accumulation (Park et al., 2017a). In another study, the transformation of *F. tataricum* with different strains of *A. rhizogenes* resulted in varied infection efficiency, growth and length of HR, and phenolic compound accumulation (Thwe et al., 2016). A similar result was obtained in this study: all the tested *A. rhizogenes* strains had the ability to induce HR formation with different infection efficiency, number and length of HRs, dry weight of roots, and phenolic compound accumulation.

Several previous studies have evaluated hairy root (HR) induction in *Ocimum basilicum* using different *Agrobacterium rhizogenes* strains and reported varying transformation efficiencies depending on the strain used. For instance, culture of *O. basilicum* HRs obtained using the A4 strain yielded a transformation frequency of 96 ± 1.99% (Pandey et al., 2022). Meanwhile, in a study on HR induction in *O. basilicum* using four different strains of *A. rhizogenes* strains, namely A4, ARqual-pTSC5, 8196, and 11325, the highest transformation frequency was obtained using A4 Conversely, in another study on HR induction in *O. basilicum* using A4, ATCC 15834, MSU, and R1000, the highest infection percentage (68.1%) was achieved using ATCC 15834 (Abadi et al., 2020). Similarly, in a study on HR culture of *O. basilicum* transformed with *A. rhizogenes* ATCC 15834, three-fold increases in the growth rate and rosmarinic acid content were noted compared with the control values (Bais et al., 2002). However, in the present study, the highest infection efficiency was achieved using the R1601 strain. Moreover, variations in the susceptibility of *O. basilicum* foliar explants to different *A. rhizogenes* strains may be attributed to differences in the degree of virulence of these strains, expression patterns of Ri transferred DNA (T-DNA) genes, and positional incorporation of T-DNA in the host genome (Bansal et al., 2014; Nourozi et al., 2014; Srivastava et al., 2016a; Pandey et al., 2022), which may further explain the differences in the transformation efficiencies of various *A. rhizogenes* strains.

The transformation of different strains of *A. rhizogenes* is an important method for increasing the production of novel compounds in plants. In the present study, we confirmed that all transgenic HRs expressed *rol* genes, as confirmed using PCR, and that the *rol* genes of the Ri plasmid in *A. rhizogenes* were responsible for HR induction (**Supplementary Figure S9**). In several plants, the highest accumulation of flavonoids and phenolics was recorded in HRs, due perhaps to the presence of *rolB*, which upregulates genes involved in secondary metabolic pathways (Weremczuk-Jeżyna et al., 2013; Thiruvengadam et al., 2014; El-Esawi et al., 2017; Bulgakov et al., 2018; Tavassoli and Safipour Afshar, 2018). Several studies have reported that HRs induced by various *A. rhizogenes* strains differ in terms of secondary metabolite production (Gupta et al., 2016; Thwe et al., 2016; Tavassoli and Safipour Afshar, 2018). In addition, they has been reported that the induction of HR is strain-specific (Tavassoli and Safipour Afshar, 2018). Several previous studies have stated that the A4 strain can be used to induce HR in medicinal plants, such as *Aesculus hippocastanum* (Zdravković-Korać et al., 2004), *Boerhaavia diffusa* (Gupta et al., 2016), *Catharanthus roseus* (Batra et al., 2004), *Gentiana macrophylla* (Tiwari et al., 2007), *Hypericum perforatum* (Vinterhalter et al., 2006), *O. basilicum* (Srivastava et al., 2016a; Abadi et al., 2020; Pandey et al., 2022), *Rhaponticum carthamoides* (Skała et al., 2015), and *Scutellaria baicalensis* (Tiwari et al., 2008). Among the different strains tested for HR induction and SM production in the plants such as *A. rugosa*, *F. tataricum*, *M. alba*, the most effective strains were *A. rhizogenes* ATCC 13333, R1000, and LBA9402, respectively (Thwe et al., 2016; Park et al., 2017a; Park et al., 2017b).

In four different *Ocimum* species, namely *O. gratissimum*, *O. basilicum*, *O. sanctum*, and *O. kilimandscharicum*, HRs induced by the A4 strain showed elevated pentacyclic triterpene levels (Pandey et al., 2022). Furthermore, in three *Ocimum* cultivars (B3, B12, and B13), HRs were induced using different *A. rhizogenes* strains, and samples induced by A4 were reported to show enhanced endogenous IAA, total phenolics, rosmarinic acid, and caffeic acid accumulation as well as increased antioxidant activity (Srivastava et al., 2016a). Abadi et al., 2020 reported than in *O. basilicum* infected with *A. rhizogenes* ATCC 15834, the HR length, number of HRs per explant, and infection frequency were the highest. In contrast, rosmarinic acid and phenolic content was higher in *O. basilicum* HRs induced using *A. rhizogenes* MAFF 03-01724 than in those induced using *A. rhizogenes* 15834 (Tada et al., 1996). This supports the results of our study, which showed that the lowest rosmarinic acid content was obtained in the ATCC 15834 infected strain. In another study, *A. rugosa* transformed with different *Agrobacterium* strains showed that ATCC 13333 strain is suitable for increasing rosmarinic acid production (Park et al., 2017b). In *F. tataricum*, the highest total phenolic content was observed in the R1000 strain. However, in the present study, the highest phenolic content was obtained in the R1601 infected strain followed by the R1200 and ATCC 13333 strains. Previous studies have reported that HRs have the ability to produce the highest content of metabolites due to higher growth rates; thus, HRs from several plant species have been used for the production of various phenolic compounds (Park et al., 2017a; Park et al., 2021). A similar result was obtained in these studies due to the higher growth rate of the R1601 infected strains which led to the highest accumulation of primary and secondary metabolites. These results show that HR induction and increase in SM levels are strain-specific in plants.

Thwe et al. (2013) analyzed the metabolic profile of *F. tataricum* HRs, and reported that the TCA cycle intermediates, such as fumaric, citric, and succinic acid, showed a positive correlation with citric acid. This result corresponded with our study results that citric acid showed a positive correlation with TCA cycle intermediates. In addition, citric acid showed a negative correlation with all phenolic compounds except *p*-coumaric acid. The same study showed that most of the phenolic compounds were negatively correlated with TCA cycle intermediates. These results show that the correlation between metabolites involved in closely related pathways was similar and proved the robustness of the results of the present study.

## Conclusions

In this study, we developed an effective method of *A. rhizogenes* mediated transformation to induce HR culture and accumulate important phenolic compounds in *O. basilicum*. Among the different strains (ATCC 13333, ATCC 15834, A4, R1000, R1200, and R1601), all enhanced HR induction and phenolic compound accumulation at different levels. However, using the method described in this study, the R1601 strain is one of the suitable strains for mass production of HR in *O. basilicum* by using the method described in this study. Based on our observations, we conclude that best *A. rhizogenes* strain — R1601, is a suitable source for enhancing phenolic compounds in *O. basilicum*. In addition, we conclude that *O. basilicum* is a promising and appropriate plant for producing phenolic compounds, especially rosmarinic acid, using the HR culture system.

## Data availability statement

The original contributions presented in the study are included in the article/**Supplementary Material**. Further inquiries can be directed to the corresponding authors.

## Author contributions

YSC and SP: conceptualization. RS, MC, HK, SY, and JK: data curation. RS, MC, HK, and JY: formal analysis. YSC and SP: investigation. RS: software and writing–original draft. RS, RR, YSC, and SP: writing–editing. YSC and SP: supervision. All authors read and approved the final manuscript.

## Funding

This research was supported by Basic Science Research Program through the National Research Foundation of Korea (NRF) funded by the Ministry of Education (2019R1A6A1A11052070, 2022R1I1A3054240, and 2022R1I1A1A01056531) funded to Yong Suk Chung, Sang Un Park, and Ramaraj Sathasivam, respectively.

## Conflict of interest

The authors declare that the research was conducted in the absence of any commercial or financial relationships that could be construed as a potential conflict of interest.

## Publisher’s note

All claims expressed in this article are solely those of the authors and do not necessarily represent those of their affiliated organizations, or those of the publisher, the editors and the reviewers. Any product that may be evaluated in this article, or claim that may be made by its manufacturer, is not guaranteed or endorsed by the publisher.
