# Supplementary material for: Effects of various Agrobacterium rhizogenes strains on hairy root induction and analyses of primary and secondary metabolites in Ocimum basilicum
Source: Front Plant Sci. 2022 Oct 17;13:983776. doi: 10.3389/fpls.2022.983776 (PMC9619037; doi:10.3389/fpls.2022.983776)
Supplement: Supplementary file 2 [file DataSheet_2.docx]

Supplementary Material

# Supplementary Figures and Tables

## Supplementary Figures


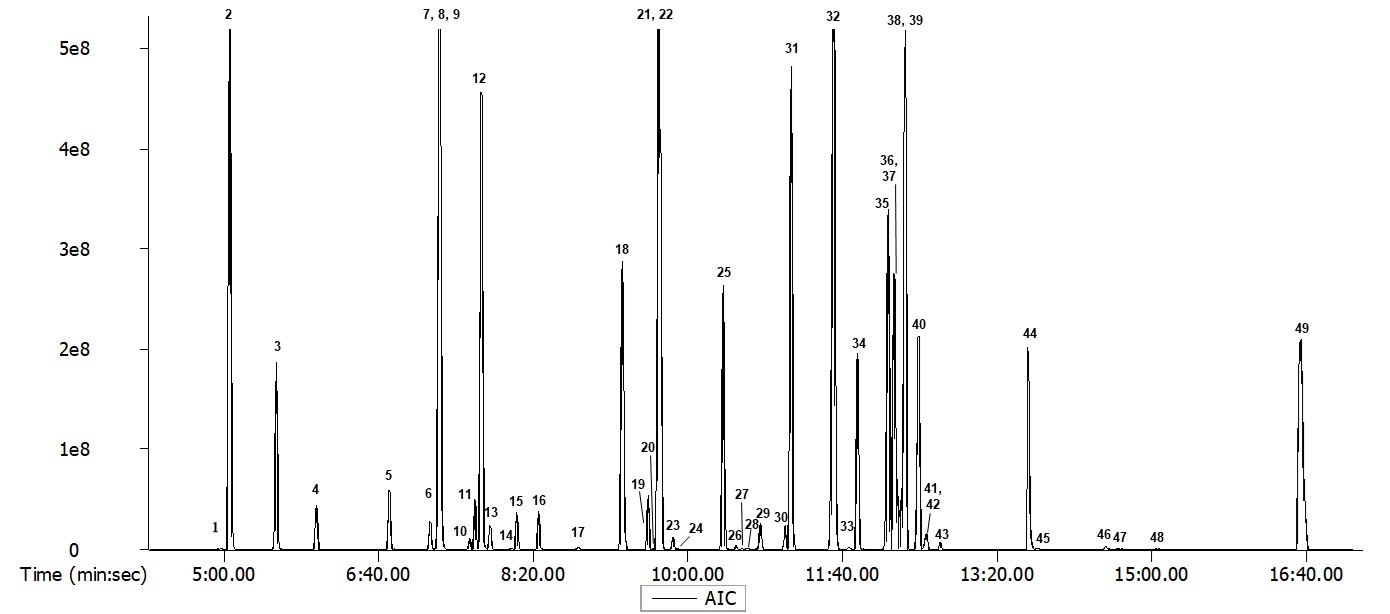


**Supplementary Figure S1┃**GC-TOF-MS analytical ion chromatogram (AIC) of hydrophilic compounds extracted from ATCC 15834. 1, Pyruvic acid; 2, Lactic acid; 3, Alanine; 4, Oxalic acid; 5, Valine; 6, Serine-1; 7, Ethanolamine; 8, Glycerol; 9, Phosphoric acid; 10, Proline; 11, Glycine; 12, Succinic acid; 13, Glyceric acid; 14, Fumaric acid; 15, Serine-2; 16, Threonine; 17, β-Alanine ; 18, Malic acid; 19, Aspartic acid; 20, Methionine; 21, Pyroglutamic acid; 22, 4-Aminobutyric acid; 23, Threonic acid; 24, Cysteine; 25, Glutamic acid; 26, Phenylalanine; 27, Xylose ; 28, Arabinose; 29, Asparagine; 30, Xylitol; 31, Ribitol; 32, Glutamine; 33, Shikimic acid; 34, Citric acid; 35, Fructose-1; 36, Fructose-2; 37, Mannose; 38, Galactose; 39, Glucose-1; 40, Glucose-2; 41, Mannitol; 42, Lysine; 43, Tyrosine; 44, Inositol; 45, Ferulic acid; 46, Tryptophan; 47, Sinapinic acid; 48, Glucose-6-phosphate; 49, Sucrose.


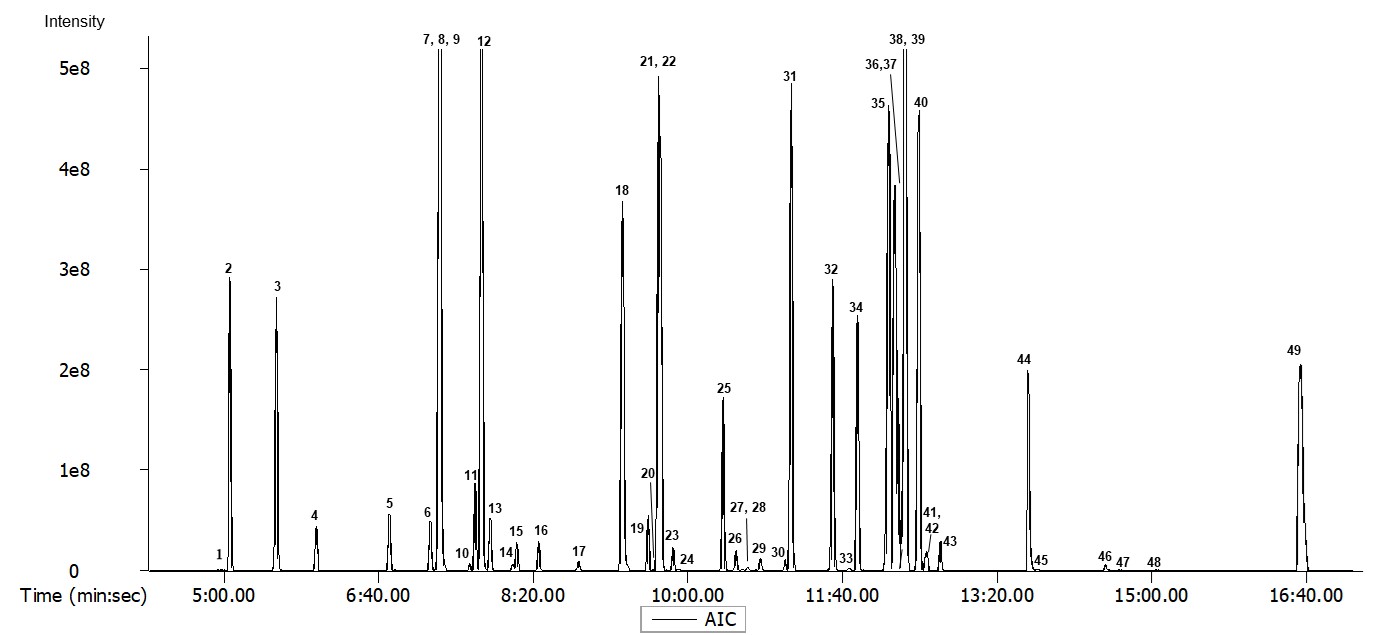


**Supplementary Figure S2┃**GC-TOF-MS analytical ion chromatogram (AIC) of hydrophilic compounds extracted from ATCC 13333. 1, Pyruvic acid; 2, Lactic acid; 3, Alanine; 4, Oxalic acid; 5, Valine; 6, Serine-1; 7, Ethanolamine; 8, Glycerol; 9, Phosphoric acid; 10, Proline; 11, Glycine; 12, Succinic acid; 13, Glyceric acid; 14, Fumaric acid; 15, Serine-2; 16, Threonine; 17, β-Alanine ; 18, Malic acid; 19, Aspartic acid; 20, Methionine; 21, Pyroglutamic acid; 22, 4-Aminobutyric acid; 23, Threonic acid; 24, Cysteine; 25, Glutamic acid; 26, Phenylalanine; 27, Xylose ; 28, Arabinose; 29, Asparagine; 30, Xylitol; 31, Ribitol; 32, Glutamine; 33, Shikimic acid; 34, Citric acid; 35, Fructose-1; 36, Fructose-2; 37, Mannose; 38, Galactose; 39, Glucose-1; 40, Glucose-2; 41, Mannitol; 42, Lysine; 43, Tyrosine; 44, Inositol; 45, Ferulic acid; 46, Tryptophan; 47, Sinapinic acid; 48, Glucose-6-phosphate; 49, Sucrose.


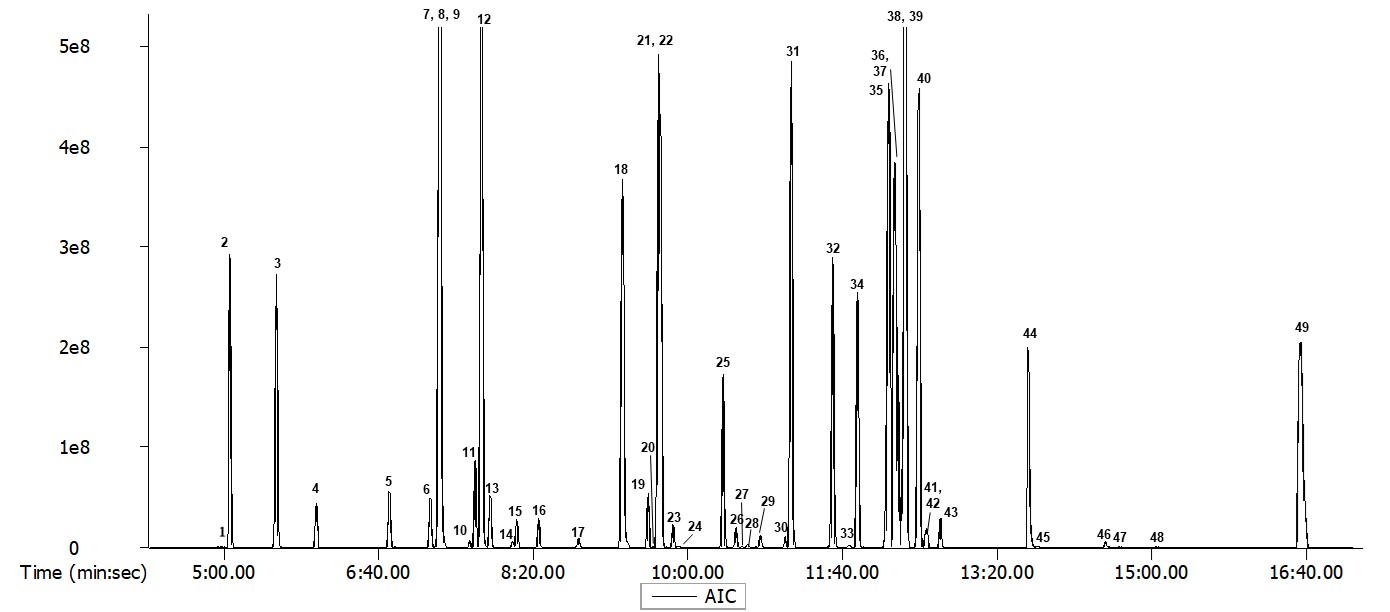


**Supplementary Figure S3┃**GC-TOF-MS analytical ion chromatogram (AIC) of hydrophilic compounds extracted from R1000. 1, Pyruvic acid; 2, Lactic acid; 3, Alanine; 4, Oxalic acid; 5, Valine; 6, Serine-1; 7, Ethanolamine; 8, Glycerol; 9, Phosphoric acid; 10, Proline; 11, Glycine; 12, Succinic acid; 13, Glyceric acid; 14, Fumaric acid; 15, Serine-2; 16, Threonine; 17, β-Alanine ; 18, Malic acid; 19, Aspartic acid; 20, Methionine; 21, Pyroglutamic acid; 22, 4-Aminobutyric acid; 23, Threonic acid; 24, Cysteine; 25, Glutamic acid; 26, Phenylalanine; 27, Xylose ; 28, Arabinose; 29, Asparagine; 30, Xylitol; 31, Ribitol; 32, Glutamine; 33, Shikimic acid; 34, Citric acid; 35, Fructose-1; 36, Fructose-2; 37, Mannose; 38, Galactose; 39, Glucose-1; 40, Glucose-2; 41, Mannitol; 42, Lysine; 43, Tyrosine; 44, Inositol; 45, Ferulic acid; 46, Tryptophan; 47, Sinapinic acid; 48, Glucose-6-phosphate; 49, Sucrose.

**
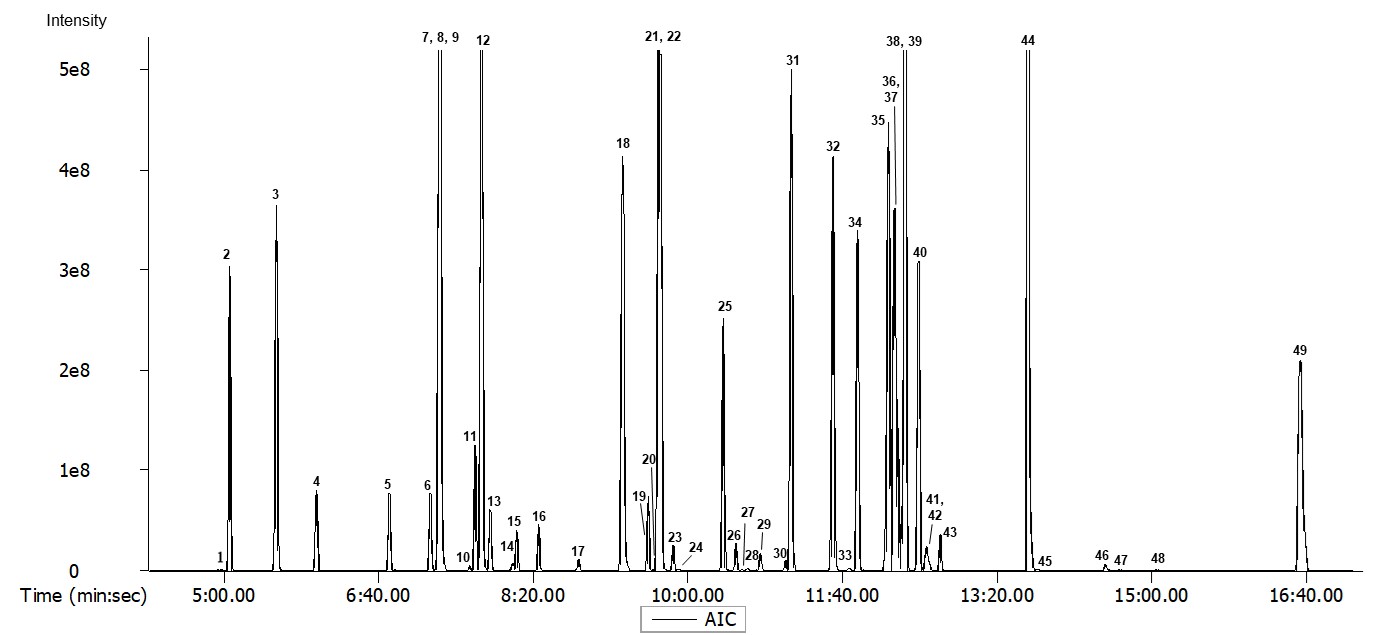
**

**Supplementary Figure S4┃**GC-TOF-MS analytical ion chromatogram (AIC) of hydrophilic compounds extracted from R1601. 1, Pyruvic acid; 2, Lactic acid; 3, Alanine; 4, Oxalic acid; 5, Valine; 6, Serine-1; 7, Ethanolamine; 8, Glycerol; 9, Phosphoric acid; 10, Proline; 11, Glycine; 12, Succinic acid; 13, Glyceric acid; 14, Fumaric acid; 15, Serine-2; 16, Threonine; 17, β-Alanine ; 18, Malic acid; 19, Aspartic acid; 20, Methionine; 21, Pyroglutamic acid; 22, 4-Aminobutyric acid; 23, Threonic acid; 24, Cysteine; 25, Glutamic acid; 26, Phenylalanine; 27, Xylose ; 28, Arabinose; 29, Asparagine; 30, Xylitol; 31, Ribitol; 32, Glutamine; 33, Shikimic acid; 34, Citric acid; 35, Fructose-1; 36, Fructose-2; 37, Mannose; 38, Galactose; 39, Glucose-1; 40, Glucose-2; 41, Mannitol; 42, Lysine; 43, Tyrosine; 44, Inositol; 45, Ferulic acid; 46, Tryptophan; 47, Sinapinic acid; 48, Glucose-6-phosphate; 49, Sucrose.

**
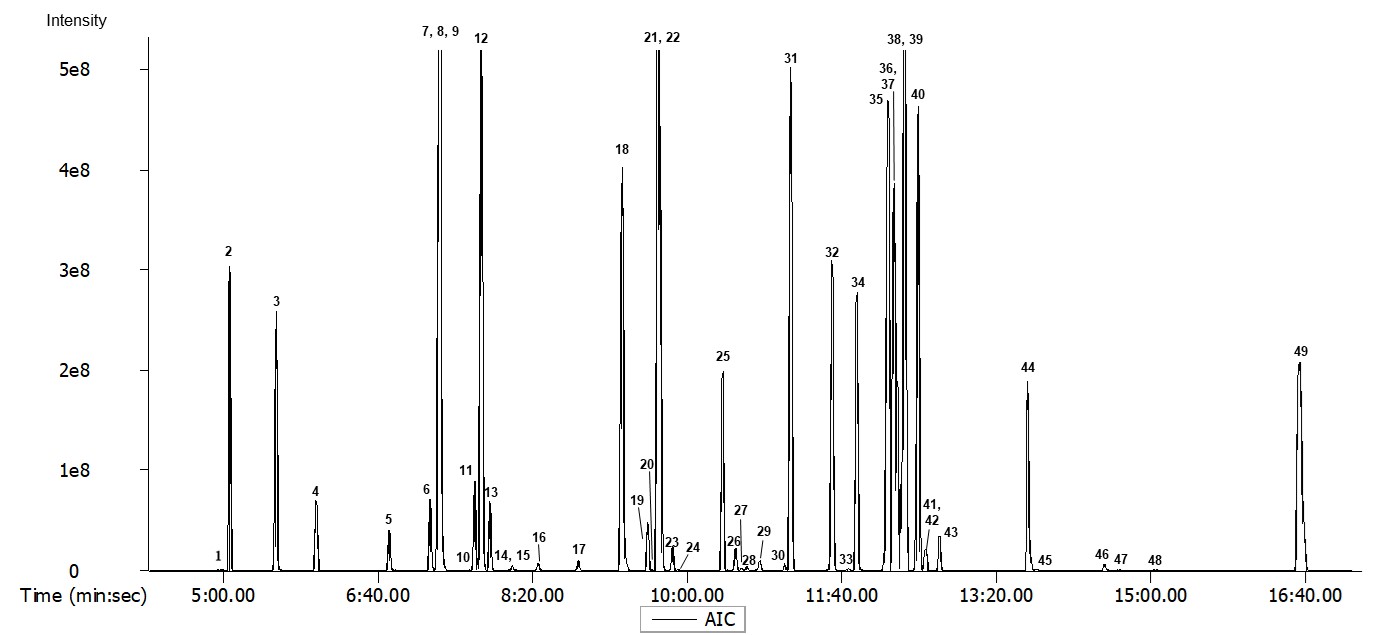
**

**Supplementary Figure S5┃**GC-TOF-MS analytical ion chromatogram (AIC) of hydrophilic compounds extracted from R1200. 1, Pyruvic acid; 2, Lactic acid; 3, Alanine; 4, Oxalic acid; 5, Valine; 6, Serine-1; 7, Ethanolamine; 8, Glycerol; 9, Phosphoric acid; 10, Proline; 11, Glycine; 12, Succinic acid; 13, Glyceric acid; 14, Fumaric acid; 15, Serine-2; 16, Threonine; 17, β-Alanine ; 18, Malic acid; 19, Aspartic acid; 20, Methionine; 21, Pyroglutamic acid; 22, 4-Aminobutyric acid; 23, Threonic acid; 24, Cysteine; 25, Glutamic acid; 26, Phenylalanine; 27, Xylose ; 28, Arabinose; 29, Asparagine; 30, Xylitol; 31, Ribitol; 32, Glutamine; 33, Shikimic acid; 34, Citric acid; 35, Fructose-1; 36, Fructose-2; 37, Mannose; 38, Galactose; 39, Glucose-1; 40, Glucose-2; 41, Mannitol; 42, Lysine; 43, Tyrosine; 44, Inositol; 45, Ferulic acid; 46, Tryptophan; 47, Sinapinic acid; 48, Glucose-6-phosphate; 49, Sucrose.

**
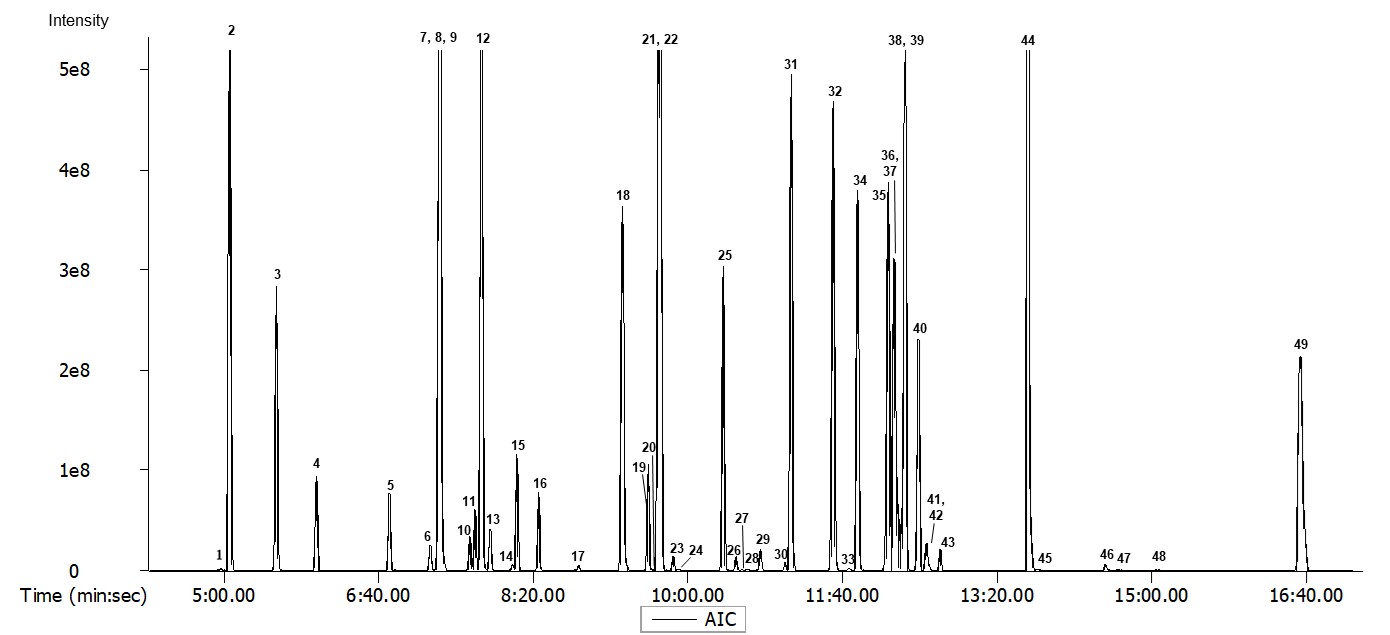
**

**Supplementary Figure S6┃**GC-TOF-MS analytical ion chromatogram (AIC) of hydrophilic compounds extracted from A4. 1, Pyruvic acid; 2, Lactic acid; 3, Alanine; 4, Oxalic acid; 5, Valine; 6, Serine-1; 7, Ethanolamine; 8, Glycerol; 9, Phosphoric acid; 10, Proline; 11, Glycine; 12, Succinic acid; 13, Glyceric acid; 14, Fumaric acid; 15, Serine-2; 16, Threonine; 17, β-Alanine ; 18, Malic acid; 19, Aspartic acid; 20, Methionine; 21, Pyroglutamic acid; 22, 4-Aminobutyric acid; 23, Threonic acid; 24, Cysteine; 25, Glutamic acid; 26, Phenylalanine; 27, Xylose ; 28, Arabinose; 29, Asparagine; 30, Xylitol; 31, Ribitol; 32, Glutamine; 33, Shikimic acid; 34, Citric acid; 35, Fructose-1; 36, Fructose-2; 37, Mannose; 38, Galactose; 39, Glucose-1; 40, Glucose-2; 41, Mannitol; 42, Lysine; 43, Tyrosine; 44, Inositol; 45, Ferulic acid; 46, Tryptophan; 47, Sinapinic acid; 48, Glucose-6-phosphate; 49, Sucrose.


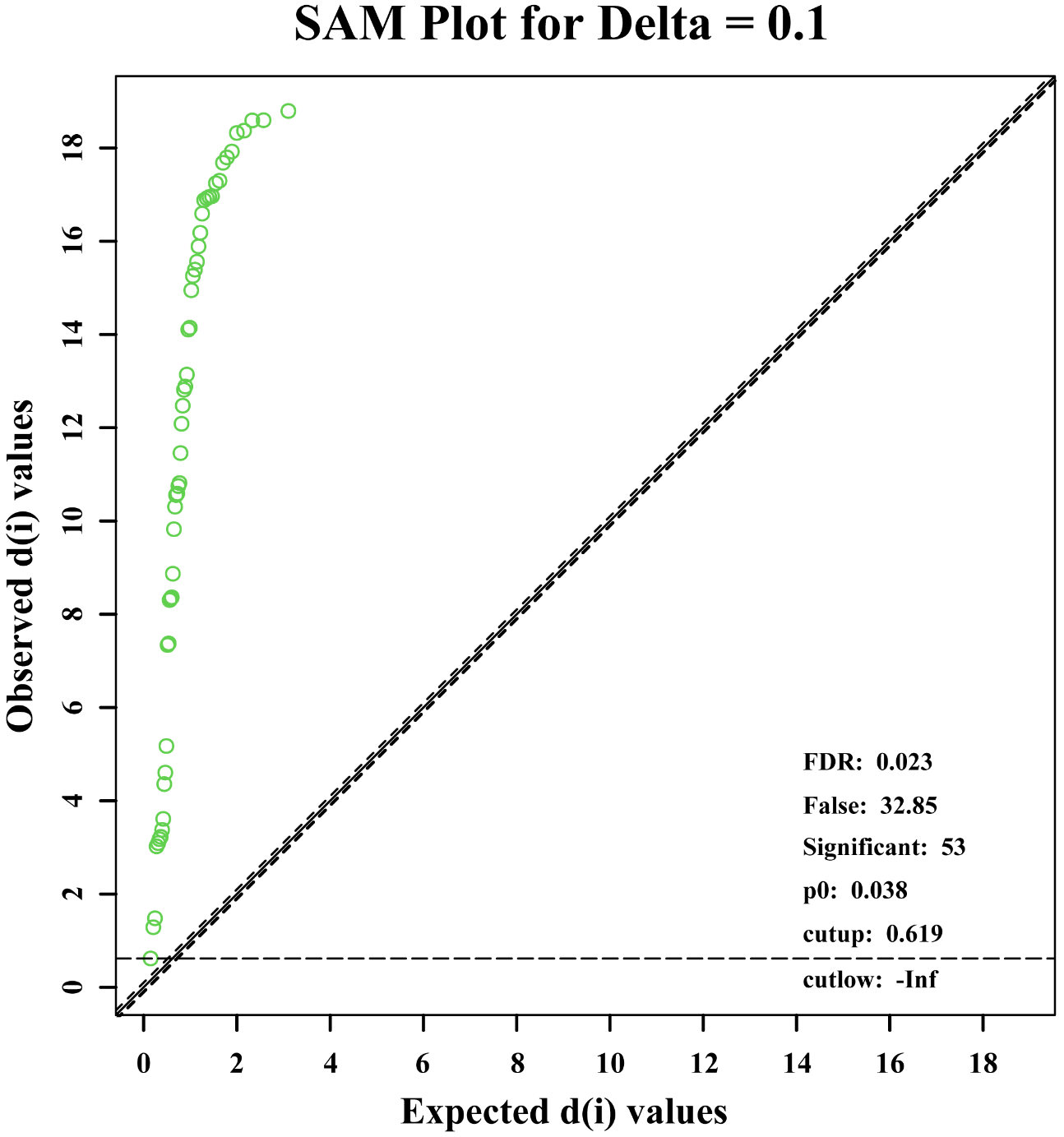


**Supplementary Figure S8┃**The important metabolites identified through SAM.


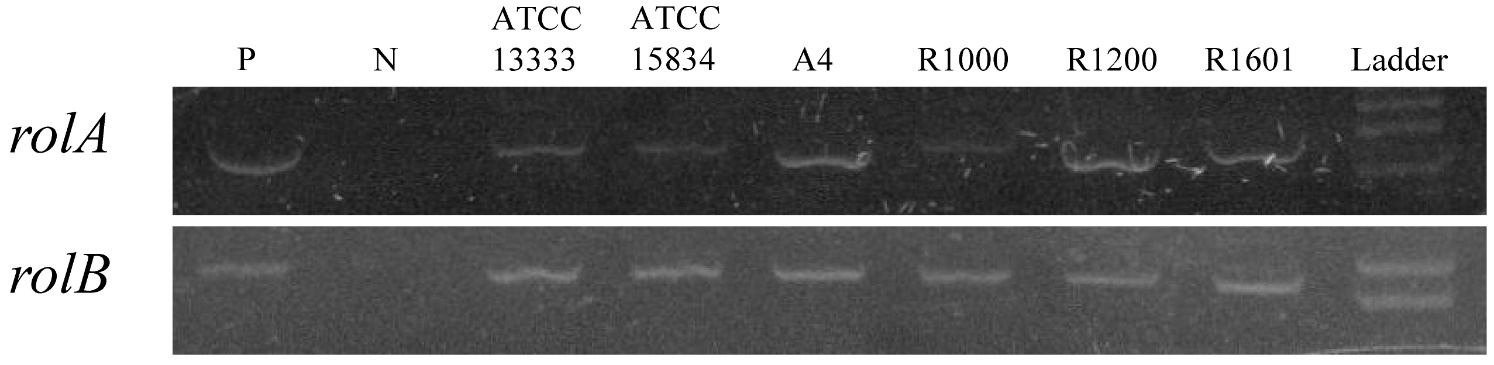


**Supplementary Figure S9┃**PCR analysis of rol A (304 bp) and rol B (797 bp) in *O. basilicum* hairy root induced by different *Agrobacterium rhizogenes* strains. 1kb ladder was used in this study. P – Positive control, N – Negative control.
